# Transvaginal Ultrasound Findings Predicting Prolonged Pregnancy in Cases of Prolapsed Fetal Membrane: A Retrospective Study

**DOI:** 10.3390/jcm14051592

**Published:** 2025-02-26

**Authors:** Tomohiro Kondo, Hiroyuki Tsuda, Eri Tsugeno, Yumi Nakamura, Yumiko Ito, Atsuko Tezuka, Tomoko Ando

**Affiliations:** Department of Obstetrics and Gynecology, Japanese Red Cross Aichi Medical Center Nagoya Daiichi Hospital, Nagoya 453-8511, Japan

**Keywords:** cervical cerclage, preterm birth, prolapsed fetal membrane, prolonged pregnancy

## Abstract

**Background/Objectives**: Fetal membrane prolapse can occur due to advanced cervical insufficiency. We investigated the yet unclear predictors of prolonged pregnancy in women with prolapsed fetal membranes. **Methods**: This retrospective observational study included 100 pregnant women with prolapsed fetal membranes between November 2017 and March 2023. We examined the correlation between transvaginal ultrasound findings at the time of admission and the duration of prolonged pregnancy, which was defined as the period from admission to delivery. We defined five transvaginal ultrasound indices: (1) width of the external os, (2) maximum width of the prolapsed fetal membrane, (3) distance from the external os to the presenting part of the fetus, (4) thickness of the posterior uterine lip, and (5) morphology of the prolapsed fetal membrane. **Results**: Women who underwent cervical cerclage comprised the cerclage group (*n* = 17), while those who underwent conservative management comprised the non-cerclage group (*n* = 83). The pregnancy period was significantly longer in the cerclage group than in the non-cerclage group (81.4 days vs. 9.1 days, *p* < 0.001). Multiple regression analysis revealed that type A morphology was a significant factor for prolonged pregnancy in the non-cerclage group (*p* < 0.05), which was significantly associated with a prolonged pregnancy period of over 7 days (*p* = 0.037). **Conclusions**: In cases of prolapsed fetal membranes, cerclage is challenging because of the high risk of iatrogenic preterm rupture of the membrane; however, if successful, a significant prolongation of the pregnancy period can be obtained. Morphological evaluation using ultrasonography is simple and easy to understand and correlates well with pregnancy outcomes, making it very useful.

## 1. Introduction

Fetal membrane prolapse is the protrusion of the fetal membranes through the cervix into the vagina, indicating advanced cervical insufficiency [1]. The American College of Obstetricians and Gynecologists defines cervical insufficiency as the inability of the uterine cervix to retain pregnancy in the second trimester in the absence of clinical contractions, labor, or both [2]. It contributes significantly to preterm birth and pregnancy loss in the second trimester and is associated with poor perinatal outcomes, even with advanced neonatal care [3]. The incidence of a short cervix ranges from approximately 2 to 6%, and the incidence of prolapsed fetal membranes is likely lower [4].

Prolapsed fetal membranes suggest imminent delivery, especially in the absence of intervention. Furthermore, in such cases, exposure of the membranes to vaginal flora may predispose them to infection or rupture. Fetal membrane prolapse leads to intra-amniotic infection in approximately 8–52% and inflammation in 81% of cases, as determined by amniocentesis [5,6]. Intra-amniotic inflammation with cervical insufficiency carries a 50% risk of preterm delivery within 7 days [5].

Emergency cerclage has demonstrated superiority over expectant management in cases of cervical insufficiency [7,8]. Previous studies have demonstrated cervical cerclage as a therapeutic strategy in fetal membrane prolapse [1,9]. Ocal et al. revealed that the prolongation of pregnancy between cerclage and bedrest groups in cases of prolapsed fetal membranes was 42.3 ± 34 and 17.9 ± 22 days, respectively [1]. In a meta-analysis evaluating cerclage for cervical dilation ≥2 cm and/or prolapsed fetal membranes before 28 weeks of gestation, cerclage was associated with improved overall survival compared with expectant management (73% vs. 36%; RR 1.6, 95% confidence interval [CI] 1.2 to 2.1) and was associated with a significant increase in the mean gestational age at birth from 25.4 weeks to 30.5 weeks, regardless of cervical dilation and gestational age at diagnosis [10]. In the only randomized trial, cerclage reduced the rate of preterm birth before 34 weeks of gestation compared to bed rest (54% vs. 100%) in cases of cervical insufficiency [11].

However, in clinical practice, cervical cerclage for prolapsed fetal membranes is sometimes extremely difficult. Prolapsed fetal membranes lead to iatrogenic preterm rupture of membranes (PROM) in approximately 65% of cases [12,13] and consequent avoidance of cervical cerclage. In such cases, most pregnant women deliver within a few weeks, but in some cases, expectant management, such as bed rest, can extend the gestational period by several weeks or more [1,9]. Only a few studies have examined the predictors of prolonged pregnancies in patients with prolapsed fetal membranes. This study aimed to compare cerclage vs. no cerclage in a prolonged pregnancy period and investigate the association between ultrasound characteristics and prolonged pregnancy period in women with fetal membrane prolapse.

## 2. Materials and Methods

### 2.1. Study Design and Participants

This was a retrospective observational study of pregnant women transferred to our hospital between November 2017 and March 2023 because of prolapsed fetal membranes. In all cases, transvaginal ultrasonography (Voluson E10, GE Healthcare, Tokyo, Japan) was performed with an empty bladder upon admission to evaluate the status of the prolapsed fetal membrane and dilatation of the cervical os by an experienced obstetrician. The depth and direction of insertion of the transvaginal probe were adjusted to delineate the entire length of the cervix. The force of the probe was regulated to prevent the cervix from being compressed. Subsequently, the image of the cervix was enlarged sufficiently on the screen while adjusting the frequency and gain for a clear contrast of the cervix. The detection of advanced fetal membranes pushing the external uterine orifice and opening it completely was diagnosed as a prolapsed fetal membrane. Our treatment strategy for patients with prolapsed fetal membranes is as follows. First, cervical cerclage is indicated for pregnancies of <25 weeks. Second, the McDonald technique was employed when cerclage was performed. Third, cerclage can be indicated if the cervix is fully visible by the vaginal speculum. However, if a patient does not wish to undergo cerclage after a detailed explanation of its advantages and disadvantages (especially the risk of PROM), expectant management is performed. Finally, if the membrane completely prolapsed into the vagina and the cervix was not visible through the vaginal speculum, expectant management was selected. However, even under such circumstances, after explaining that the risks and disadvantages of cerclage are very high, and if desired, cerclage was performed. For expectant management, the patients were treated with tocolytic agents (ritodrine hydrochloride or magnesium sulfate) for the required period. Antibiotics (azithromycin 1 gm orally once + sulbactam/ampicillin 3 gm intravenously every 12 h for 7 days) were also administered if blood tests revealed elevated levels of inflammatory markers. The McDonald technique was used, with a curved needle loaded with a large-caliber, nonabsorbable synthetic suture (monofilament) inserted into the cervix in the 12 o’clock direction. Four deep bites of a purse-string suture were taken circumferentially around the cervix (in the order of 9, 6, 3, and 12 o’clock positions) in a counterclockwise direction, as safely as possible (close to the internal os). The two ends of the sutures were cut by tight tying. In this study, patients were divided into two groups: a cerclage group that underwent cerclage and a non-cerclage group that underwent expectant management. If clinical chorioamnionitis was strongly suspected, the diagnosis was based on Lencki’s criteria [12,13], and the patient delivered promptly and was excluded from the study. There were no cases of twin pregnancies or those that required imminent delivery due to maternal or fetal complications, such as severe pre-eclampsia or severe fetal growth restriction. The following data were extracted from the medical and midwifery records: maternal age, gestational weeks at admission, cervical length at admission, pre-pregnancy body mass index, parity, use of assisted reproductive technology, history of preterm birth, laboratory data, ultrasound findings at admission, and weeks of gestation at delivery. Prolonged pregnancy was defined as the period from the date of maternal transfer to the hospital to the date of delivery. No patients received vaginal progesterone or pessary insertion. The use of these data was approved by the Institutional Review Board of the Japanese Red Cross Nagoya Daiichi Hospital (2023-250). Patient informed consent was waived as this study used anonymous clinical data.

### 2.2. Ultrasound Findings

**Figure 1:**
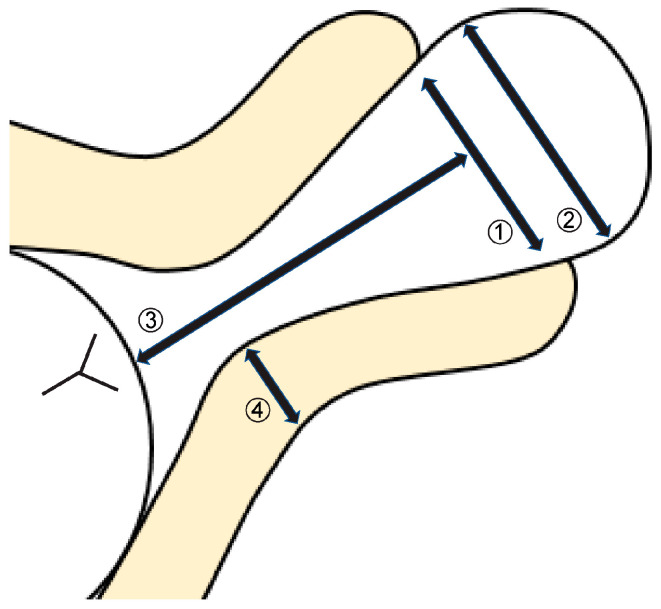
Definition of transvaginal ultrasound findings. (1) Width of the external os; (2) maximum width of prolapsed fetal membrane; (3) distance from the external os to the presenting part of the fetus; (4) thickness of the posterior uterine lip. This was measured at the location of the internal cervical os.

**Figure 2:**
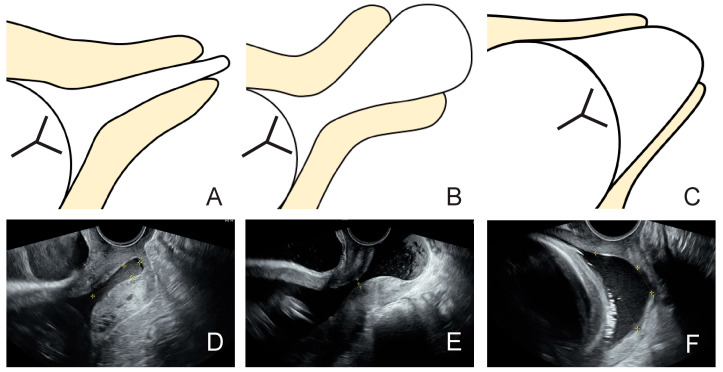
Morphologic classification of prolapsed fetal membrane. (**A**) We defined type A: the effacement of the cervix has not progressed, and the dilation of the external cervical os is wider than the width of the prolapsed fetal membrane. (**B**) We defined type B: the effacement of the cervix has not progressed, and the dilation of the external cervical os is smaller than the width of the prolapsed fetal membrane. (**C**) We defined type C: the effacement of the cervix has progressed, and the prolapsed fetal membrane has formed a dome shape. (**D**–**F**) are examples of type A, B, and C, respectively.

In the non-cerclage group, we examined the correlation between transvaginal ultrasound findings at the time of admission and the duration of prolonged pregnancy. Five new transvaginal ultrasound indices for this study were developed based on the Bishop score [14]: (1) width of the external os, (2) maximum width of the prolapsed fetal membrane, (3) distance from the external os to the presenting part of the fetus, (4) thickness of the posterior uterine lip, and (5) morphology of the prolapsed fetal membrane, which was classified into three categories (types A–C) (Figure 1 and Figure 2).

### 2.3. Statistical Analysis

All statistical analyses were performed using EZR software (v. 1.37, Saitama, Japan). The Shapiro–Wilk test was used to analyze the normality of the data. Continuous variables between the two groups were compared using Student’s *t*-test. The Mann–Whitney U test was used for non-parametric comparisons. Nominal data were analyzed using Fisher’s exact test. When comparing the three groups, we used a one-way analysis of variance and Bonferroni’s multiple comparison test for post hoc analysis. In multivariate analysis, body mass index, parity, ultrasound findings, and gestational age at admission were selected as variables associated with prolonged pregnancy; additionally, assisted reproductive technology and history of preterm delivery—which are generally known as risk factors for preterm delivery—were selected as clinically important variables. A receiver-operating characteristic curve was generated to calculate the cut-off values for a prolonged pregnancy period of <7 days. Statistical significance was set at *p* < 0.05.

## 3. Results

Our hospital is a tertiary center that accepted 1352 maternal transfers between November 2017 and March 2023, of which 100 cases were of prolapsed fetal membranes. This study included these 100 patients. Of these, 17 were in the cerclage group, and 83 were in the non-cerclage group (Figure S1). After the exclusion of 8 of the 83 patients in the non-cerclage group (5 with clinical chorioamnionitis, 2 with lack of ultrasound images, and 1 with loss of follow-up), the group finally comprised 75 patients. In the cerclage group, only two women (11.8%) delivered within 4 weeks; the remaining fifteen (88.2%) were able to elongate the pregnancy for more than 4 weeks, and nine women (52.9%) delivered at term (Figure S1). In contrast, in the non-cerclage group, 53 patients (70.7%) delivered within 7 days. The prolonged pregnancy period was significantly longer in the cerclage group than in the non-cerclage group (81.4 days vs. 9.1 days, *p* < 0.001).

In 75 cases, in which ultrasound data were available for the non-cerclage group, the maximum width of the prolapsed fetal membrane was significantly negatively correlated with a prolonged pregnancy period (r = −0.35, 95% CI −0.535 to −0.134, *p* = 0.002), and the distance from the external os to the presenting part of the fetus was significantly positively correlated with a prolonged pregnancy period (r = 0.304, 95% CI 0.083 to 0.497, *p* = 0.008). The width of the external os and the thickness of the posterior uterine lip were not significantly correlated with a prolonged pregnancy period (r = −0.213, 95% CI −0.42 to 0.014, *p* = 0.066 and r = −0.123, 95% CI −0.341 to 0.106, *p* = 0.291, respectively). Morphological classification using ultrasound revealed that type A had a significantly longer pregnancy period (31.4 days) than types B (5.0 days) and C (9.6 days) (*p* < 0.001 and *p* < 0.001, respectively).

Multiple regression analysis of the duration of prolonged pregnancy is shown in Table 1. Nulliparity and type A were significant factors associated with prolonged pregnancy. Ultrasound findings other than morphological classification were not significant factors for prolonged gestational age.

The factors associated with delivery within 7 days of admission (ultrasound examination) are shown in Table 2. The width of the external os, width of the prolapsed fetal membrane, and morphologic classification (types B and C) were significant risk factors for delivery within 7 days.

**Figure 3:**
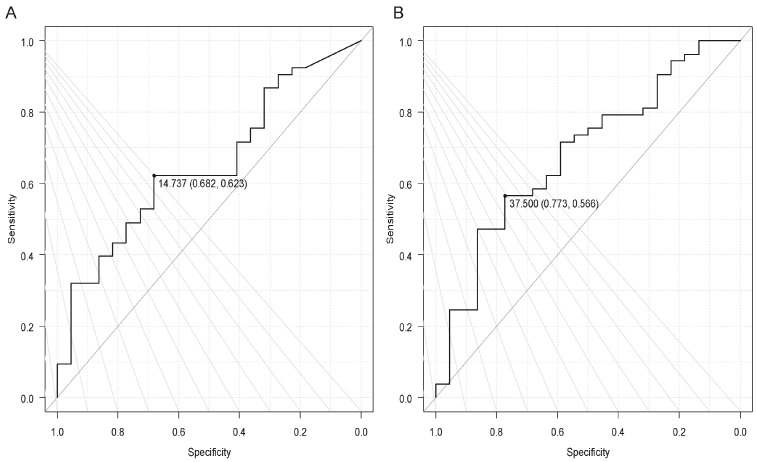
A receiver-operating characteristic (ROC) curve to calculate cutoff values for a prolonged pregnancy period of <7 days. (**A**) The width of the external os: the cutoff value was 14.7 mm. The area under the curve (AUC) of 0.651 (95% confidence interval [CI]: 0.520−0.784), sensitivity 0.623, and specificity 0.682. (**B**) The maximum width of the prolapsed fetal membrane: the cutoff value was 37.5 mm. AUC of 0.683 (95% CI: 0.549–0.816), sensitivity 0.566, and specificity 0.773.

The receiver-operating characteristic cut-off values for prolonged pregnancy within 7 days were as follows: the width of the external os was 14.7 mm; the maximum width of the prolapsed fetal membrane was 37.5 mm (Figure 3).

## 4. Discussion

This study revealed that most pregnant women who underwent cerclage were able to continue their pregnancies for >4 weeks, and more than half of them delivered at term. Conversely, in those who did not undergo cerclage, we examined the association of various ultrasound findings with the duration of prolonged pregnancy and found that the type A pattern was associated with a significantly longer duration of pregnancy in a multivariate logistic analysis, and the non-type A pattern, width of the external os, and width of the prolapsed fetal membrane were significantly associated with delivery within 7 days.

According to some reports, successful cerclage in cases of prolapsed fetal membranes can prolong the gestational period and improve the prognosis of infants [1,3,9]. Althuisius et al. [11] examined 23 pregnant women before 27 weeks of gestation with fetal membrane prolapse and revealed that the mean prolonged pregnancy period was 54 days in the emergency cerclage group and 20 days in the bed rest-only group (*p* = 0.046), which is consistent with our present data. However, cerclage cannot be performed in cases with markedly progressive fetal membrane prolapse. If cerclage is performed in these cases, rupture of the membranes intraoperatively or in the immediate postoperative period is a major concern [15]. If cerclage placement is attempted in this setting, we should recognize some tips: placing the patient in a steep Trendelenburg position, administering tocolytic drugs (e.g., nitroglycerin), backfilling the bladder in 250 mL increments through a bladder catheter [16], pushing the fetal membranes into the cervix with a balloon [17], and ultrasound-guided amniotic fluid reduction to decrease pressure on the prolapsed fetal membrane [18]. Until now, in most cases in which the cervix was not visible, we avoided cerclage. In the future, we intend to actively perform cerclage using these tips to improve pregnancy outcomes.

To date, no guidelines regarding the severity of cervical insufficiency have been established. Prolapsed fetal membranes are considered a severe form of cervical insufficiency [19], and in those cases, the duration of prolonged pregnancy is generally shorter. There are only a few reports on clinical features that predict the duration of prolonged pregnancy. Therefore, we focused on ultrasound findings in cases of prolapsed fetal membranes in which cerclage could not be performed and evaluated its association with the duration of prolonged pregnancy. Roman et al. proposed staging criteria (stages 1–5) for cervical changes at <24 weeks to predict preterm birth and recommended management according to each stage [20]. Park et al. [21] reported a new quantification system for assessing the degree of cervical insufficiency using a combination of ultrasonography and physical examination. They classified the patients into three stages (stages I–III) based on physical and ultrasound examinations, examined preterm delivery rates and prolonged gestational age, and reported that these findings showed significant correlations [21]. However, all patients included in their study underwent therapeutic cerclage, which was different from that in the present study. In this study, type A morphologic classification was a significant factor for prolonged pregnancy, especially of >7 days. Moreover, it is noteworthy that 67% of the type A cases achieved a prolonged pregnancy period of >7 days, and the average prolonged pregnancy period was 31 days. In cases of prolapsed fetal membranes, the timing of betamethasone administration should be considered because of impending delivery. However, in type A, a high probability that the pregnancy will continue for >7 days, the patient may be able to wait for betamethasone administration, which can be an important guide for administering betamethasone at the appropriate time. Morphologic classification in cases of prolapsed fetal membranes by ultrasound is a completely new method that has never been used before and is unique and intuitive. We believe that this is one of the strengths of our study and will be useful in daily clinical practice in the future.

In this study, the maximum width of the prolapsed fetal membrane showed a significant negative correlation with a prolonged pregnancy period, and the distance from the external os to the presenting part of the fetus showed a significant positive correlation with a prolonged pregnancy period. Although both correlations exhibited no significance in the multivariate logistic analysis, we speculate that the type A finding suggests that the maximum width of the prolapsed fetal membrane is small and the distance from the external os to the presenting part of the fetus tends to be long because it is tubular (Figure 2). Therefore, in the multivariate logistic analysis, these factors were considered to have offset each other, yielding no significant differences. Based on the above, we concluded that the type A finding is the best predictor of prolonged pregnancy period and delivery over 7 days and more practical than other ultrasound findings.

When performing transvaginal ultrasound, as the cervix effaces, the relationship between the lower uterine segment and the axis of the cervical canal also changes, represented by the shape of the letters “T”, “Y”, “V”, and “U”, in that order [22]. In that case, the “T” shape represents the normal pattern, whereas “U” represents almost complete effacement and signifies the highest risk for spontaneous preterm birth. Type A pattern in this study corresponds to the “T” shape, in which the fetal membrane prolapses but no cervical effacement occurs, and thus the longest pregnancy extension is presumably achieved. However, type B is associated with a large fetal membrane prolapse due to increased intrauterine pressure, and type C is associated with progressive cervical effacement (so-called “U” shape), resulting in an earlier delivery. As described above, in cases of prolapsed fetal membranes, the cervix is thought to progress from type A to B and then to C.

The strengths of this study are as follows. First, it was conducted at a single center; therefore, pregnancy management and treatment policies were uniform, and a certain quality of practice was maintained. Second, high-quality ultrasound examinations were performed by experienced obstetricians. Finally, the novel and unambiguous observation that type A pattern is significantly associated with a prolonged pregnancy period has practical implications in clinical practice. As discussed above, there are several reports on clinical features that predict the duration of prolonged pregnancy in women with prolapsed fetal membranes; however, all patients included in that study underwent therapeutic cerclage [20,21]. To the best of our knowledge, there are no reports regarding this issue in non-cerclage cases.

This study has several limitations. First, there were no data on the history of miscarriage or very preterm delivery, which are strong risk factors for spontaneous preterm birth during the current pregnancy. Second, the decision to perform cerclage was not based entirely on medical indications alone but rather on a number of patient wishes. Therefore, our study may have been subject to a selection bias. A deeper analysis is needed to elucidate how these limitations specifically impact the results. Finally, we did not use vaginal progesterone supplementation in any of the cases. In cases of a short cervix, combined therapy (vaginal progesterone supplementation after cerclage) appears to improve outcomes compared with cerclage alone [23,24]. Therefore, further research is needed in the future.

In the future, based on the results of this study, we aim to clarify the following in patients with prolapsed fetal membranes: (1) the duration of prolonged pregnancy after cerclage using the ultrasound indexes obtained in this study, and (2) whether the type A pattern changes over time to type B and subsequently to type C.

## 5. Conclusions

This study revealed that most pregnant women who underwent cerclage, even if they had prolapsed fetal membranes, were able to continue their pregnancies for >4 weeks, and more than half of them delivered at term. Conversely, in pregnant women who did not undergo cerclage, the type A pattern on ultrasound was associated with a significantly longer pregnancy duration in a multivariate logistic analysis. The novelty of this study is that the morphologic classification of prolapsed fetal membranes was evaluated based on ultrasound findings. Morphological evaluation by ultrasound is simple, easy to understand, and correlates well with pregnancy outcomes, making it very useful, and we believe these data provide valuable information for clinical practice.

## Figures and Tables

**Table 1:** Multiple regression analysis of the duration of prolonged pregnancy for prolapsed fetal membrane.

	Adjusted OR	95% CI	*p*-Value
Body mass index	0.623	−0.16–1.41	0.117
Nulliparity	2.399	1.36–6.67	0.007
Natural pregnancy	1.050	−8.92–11.02	0.834
History of preterm birth	7.727	−7.55–23.0	0.316
Width of external os	−0.167	−0.41–0.07	0.166
Width of prolapsed membrane	−0.024	−0.28–0.23	0.853
Distance between external os and baby	0.173	−0.08–0.43	0.173
Thickness of posterior uterine lip	−0.053	−1.13–1.02	0.921
Gestational weeks at admission	0.080	−0.07–0.23	0.298
Type of prolapsed fetal membrane			
B comparing with A	−17.469	−31.5–−3.47	0.0153
C comparing with A	−15.369	−28.11–−2.63	0.019

OR, odds ratio; CI, confidence interval.

**Table 2:** Factors for the delivery within 7 days in this study.

	Delivery < 7 Days (*n* = 53)	Delivery > 7 Days (*n* = 22)	*p*-Value
Width of external os (mm)	19.9 (0–93.5)	13.3 (0–51.7)	0.040
Width of prolapsed fetal membrane (mm)	38.2 (6.6–93.5)	27.0 (0–86.1)	0.013
Distance from external os to baby (mm)	15.7 (0–68.2)	24.4 (2.43–60.6)	0.067
Thickness of the posterior lip (mm)	8.50 (0–23.1)	6.74 (0.75–14.6)	0.093
Type of prolapsed fetal membrane			0.037
A	3/9 (33.3%)	6/9 (66.7%)	
B	32/41 (78.0%)	9/41 (22.0%)	
C	18/25 (72%)	7/25 (28%)	

## Data Availability

The raw data supporting the conclusions of this article will be made available by the authors on request.

## Supplementary Materials

The following supporting information can be downloaded at: https://www.mdpi.com/article/10.3390/jcm14051592/s1, Figure S1: Study population.

## Abbreviations

The following abbreviations are used in this manuscript:

PROM	Preterm rupture of membrane
CI	Confidence interval
