# Supplementary figures and images for: Transvaginal Ultrasound Findings Predicting Prolonged Pregnancy in Cases of Prolapsed Fetal Membrane: A Retrospective Study
Source: J Clin Med. 2025 Feb 26;14(5):1592. doi: 10.3390/jcm14051592 (PMC11899934; doi:10.3390/jcm14051592)

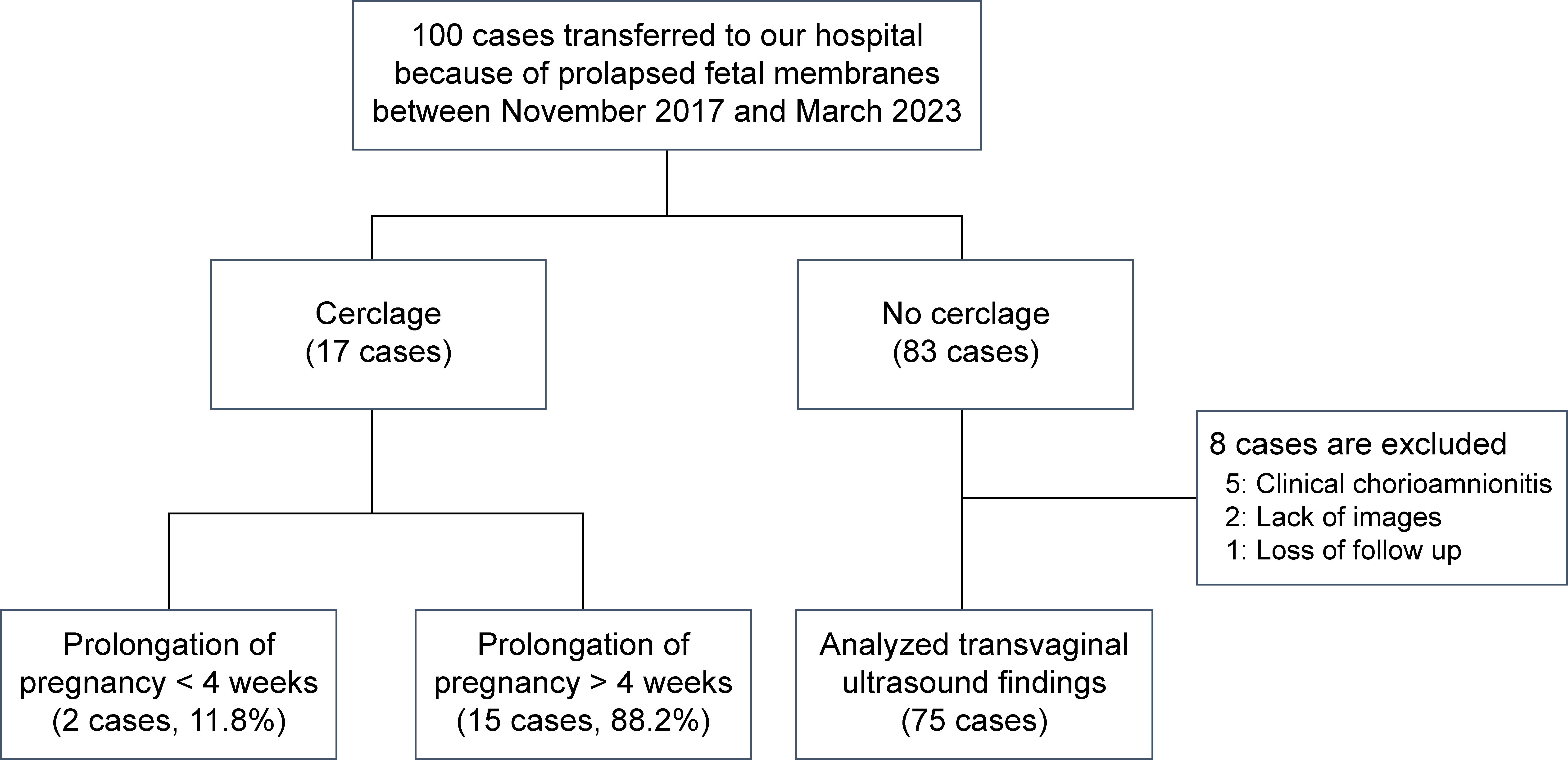

Supplement: Supplementary file 1 [file jcm-14-01592-s001.zip › Figure s1.tif]
